# Evaluation of outcome from endovascular therapy for Budd-Chiari syndrome: a systematic review and meta-analysis

**DOI:** 10.1038/s41598-022-20399-x

**Published:** 2022-09-28

**Authors:** Gauri Mukhiya, Xueliang Zhou, Xinwei Han, Dechao Jiao, Gaurab Pokhrel, Yahua Li, Sita Pokhrel

**Affiliations:** 1 The First Affiliated Hospital of Zhengzhou University, Jian She Road Erqi District, Zhengzhou, 450052 Henan China; 2 Universal College of Medical Science, Road-Ranigaon, 32900 Bhairahawa, Nepal

**Keywords:** Gastroenterology, Medical research

## Abstract

This study was performed to evaluate the outcome of endovascular intervention therapy for Budd-Chiari syndrome (BCS) and compare recanalization, transjugular intrahepatic portosystemic shunt (TIPS)/direct intrahepatic portosystemic shunt (DIPS), and combined procedure treatment. For the meta-analysis, 71 studies were identified by searching four databases. The individual studies’ samples were used to calculate a confidence interval (CI 95%), and data were pooled using a fixed-effect model and random effect model. The pooled measure and an equal-weighted average rate were calculated in all participant studies. Heterogeneity between the studies was assessed with I^2^, and T^2^ tests, and publication bias was estimated using Egger’s regression test. A total of 4,407 BCS patients had undergone an endovascular intervention procedure. The pooled results were 98.9% (95% CI 97.8‒98.9%) for a technical success operation, and 96.9% (95% CI 94.9‒98.9%) for a clinical success operation. The re-intervention rate after the initial intervention procedure was 18.9% (95% CI 14.7‒22.9%), and the survival rates at 1 and 5 years after the initial intervention procedure were 98.9% (95% CI 96.8‒98.9%) and 94.9% (95% CI 92.9‒96.9%), respectively. Patients receiving recanalization treatment (98%) had a better prognosis than those with a combined procedure (95.6%) and TIPS/DIPS treatment (94.5%). The systematic review and meta-analysis further solidify the role of endovascular intervention treatment in BCS as safe and effective. It maintains high technical and clinical success and long-term survival rates. The recanalization treatment had a better prognosis and outcome than the combined procedures and TIPS/DIPS treatment.

## Introduction

Budd-Chiari syndrome (BCS) is a rare hepatic venous disease. It presents with thrombosis, located anywhere from the hepatic veins (HV) to the suprahepatic of the inferior vena cava (IVC). The result is an outflow obstruction of hepatic veins^1,2^. The obstruction of BCS is classified as primary or secondary depending on the site of hepatic vein obstruction. The obstruction site can be a thrombus inside the vein or outside the vein due to compression with tumors^3^. The pathogenesis of BCSs remains unclear, but some known risk factors include myeloproliferative neoplasm, use of oral contraceptive drugs, and coagulation factors^4,5^. An HV outflow obstruction might cause centrilobular congestion and hepatocyte necrosis. If not treated in time, this can lead to liver cirrhosis, portal hypertension, and ascites. The clinical manifestations of BCS are abdomen pain, hepatomegaly, and ascites^6,7^. The cause and type of BCS vary by geographical regions; in Western countries, the common cause is HV obstruction, but IVC obstruction is predominate in Eastern countries^8,9^. Most frequent cause of BCS is thrombophilia, which is detected in more than 84% of patients with BCS^10,11^. The European Association for the Study of the Liver has recommended a step-wise therapeutic algorithm for BCS. The algorithm depends on treatment response, medical therapy with anticoagulant drugs, angioplasty, stent implantation, thrombolysis, transjugular intrahepatic portosystemic shunt (TIPS), and liver transplantation^12^. The progressive improvement in radiological intervention therapy in the past two decades has provided a better survival rate for BCS treatment with an intervention procedure than other treatment modalities. Recently, there has been an increase in the number of BCS patients managed with endovascular intervention therapy.

This systematic review and meta-analysis aimed to evaluate the technical and clinical success rates of endovascular intervention operation and re-intervention (including re-occlusion, re-stenosis stent, and shunt dysfunctions). We evaluated the success rates after the initial intervention procedure and the survival rate at 1 and 5 years after the initial intervention procedure. Moreover, this review compares the difference in outcome between recanalization, TIPS/DIPS, and a combined procedure (recanalization and TIPS/DIPS).

## Methods

### Search strategy

The PubMed, EMBASE, Cochrane Library and Science-Direct databases were searched for relevant published papers. The last search was performed on May 28, 2021. The following search terms were used: Budd-Chiari syndrome, hepatic venous outflow obstruction, hepatic vein stenosis, hepatic vein occlusion, hepatic vein obstruction, supra-hepatic IVC obstruction, membranous obstruction of IVC, endovascular treatment, interventional procedure, transjugular intrahepatic portosystemic shunt (TIPS), direct intrahepatic portosystemic shunt (DIPS), percutaneous transluminal balloon angioplasty(PTBA), percutaneous transluminal angioplasty (PTA) of the hepatic vein, vascular recanalization of the hepatic vein, vascular stent implantation in the hepatic vein, and vascular stent implantation in IVC.

### Selection criteria

**Figure 1:**
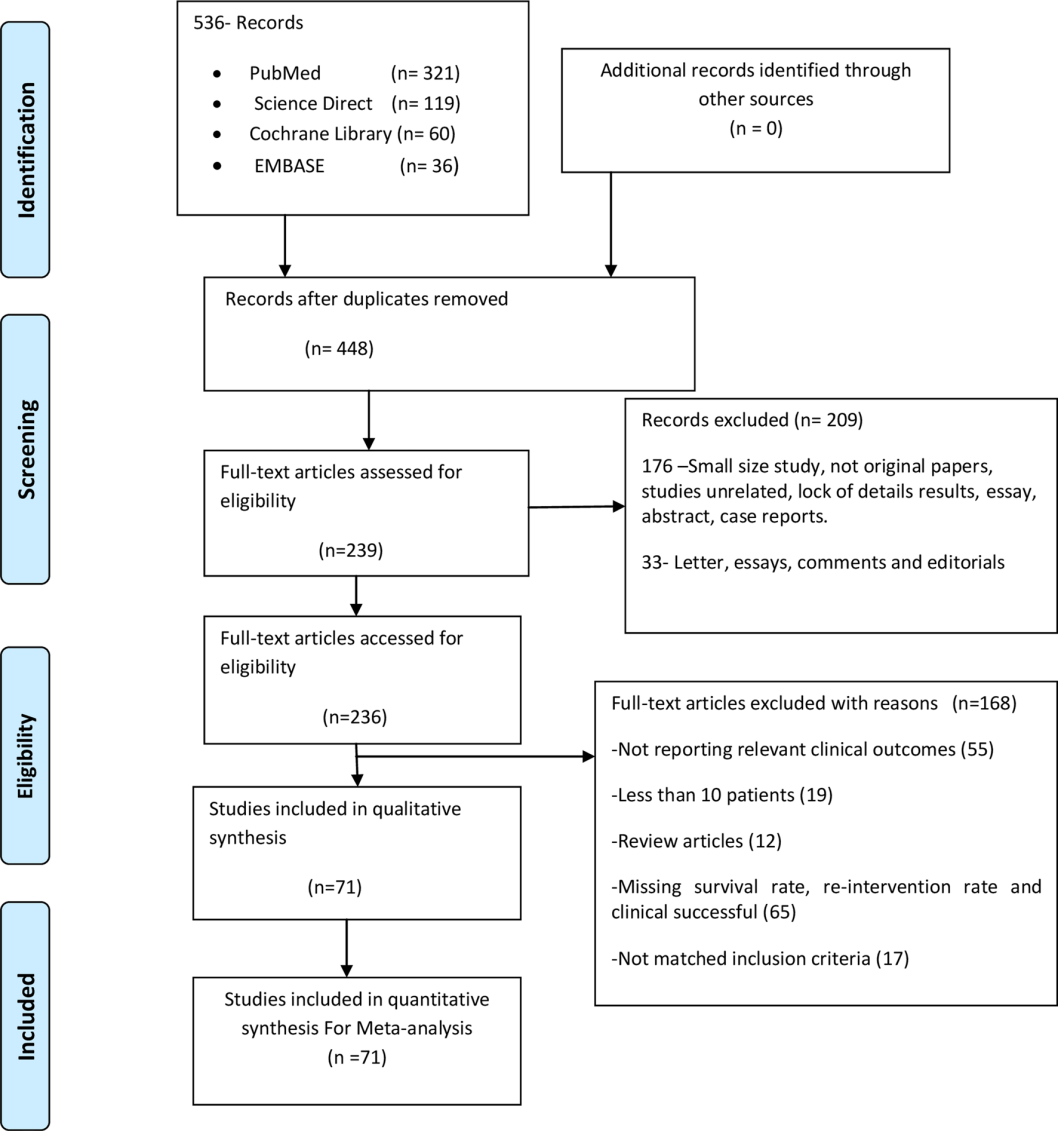
PRISMA flowchart of studies selection.

The following criteria were used to determine those studies to include: (1) study had more than ten case participants; (2) retrospective studies, prospective studies, including case series, and case–control studies were eligible; (3) all participants of any age, race, origin with a diagnosis of BCS; (4) full article papers with detailed information and statistical results of intervention treatment; and (5) there were no publication data, publication language or publication status restrictions. Exclusion criteria were: (1) duplicates studies; (2) studies that were not original papers; (3) case reports; (4) comments, (5) essays; (6) abstracts; (7) small case series; (8) not reporting relevant clinical outcomes; (9) lack of detailed results; (10) review articles; (11) less than ten patients; (12) studies unmatched inclusion criteria; (13) studies with missing survival rate, re-intervention rate or clinical success. The study selection process followed the Preferred Reporting Items for Systematic Reviews and Meta-analysis (PRISMA) guideline flowchart (Fig. 1)^13^. The PRISMA checklist is provided in (Supplementary Table 1).

### Data extraction

The following data were extracted for further analysis: (1) First author, publication year, enrollment period, country, number of BCS patients with endovascular intervention treated, age, gender, site of the obstruction (HV, IVC and combination), type of intervention procedures, technical and clinical success rate, rate of the re-intervention (re-occlusion, re-stenosis stent and dysfunction of shunt), and survival rate at 1 and 5 years.

### Quality assessment

Studies were considered higher quality if they fulfilled all the following predetermined criteria: (1) patients were admitted to the hospital; (2) the interval of enrollment and eligibility criteria was recorded; (3) the site of obstruction of BCS patients was reported; and (4) Patients were diagnosed with BCS and treated with endovascular intervention procedures.

### HV-angioplasty

When the stiff guide wire was established, a balloon dilator catheter of 12‒15 mm diameter was inserted from the right jugular vein puncture site to the obstructed part of HV/IVC via the guide wire. Next, the balloon catheter was dilated twice, and each dilatation occurred for approximately 40 s. If there was more than 30% residual stenosis on HV venography after balloon dilation then a stent was inserted in the stenosis part of the HV.

### IVC-angioplasty

Venography was performed (right femoral vein or right jugular vein) to evaluate the IVC anatomy and obstruction characteristics. Next, a guidewire with a balloon catheter (25‒30 mm) was used to dilate IVC obstructive lesions. A self-expandable metallic stent was used if the IVC narrowed immediately after balloon dilatation or more than 30% residual stenosis on IVC venography after balloon dilation.

### Combined HV and IVC angioplasty

Combined HV and IVC stenting were performed in patients having short-segment HV and IVC obstructions.

### Recanalization

Recanalization (PTA with or without stent placement) has been used in 31 (43.66%) studies with or without stent placement. In the subgroup, we analyze the technical and clinical success rate of recanalization, re-intervention treatment, and survival rate at 1 and 5 years of recanalization procedure. It was performed with balloon dilation or endovascular stent placement in the stenosis part of HV and IVC.

### TIPS/DIPS

TIPS/DIPS were used in 17 (23.94%) studies. In the subgroup, we analyze the technical and clinical success rate of TIPS/DIPS, re-intervention treatment, and survival rate at 1 and 5 years of TIPS/DIPS procedure. This was performed in symptomatic patients with non-recanalization HV obstruction with small collaterals draining into IVC, portal hypertension, refractory ascites, variceal bleeding, and long segment obstruction HV. DIPS usually used in failed TIPS, occluded three major HVs and anomalies of HVs.

### Recanalization and TIPS/DIPS (combined procedures)

Recanalization (PTA with or without stent placement) and TIPS/DIPS were used in 23 (32.39%) studies. We analyze the technical and clinical success rate, re-intervention treatment, and survival rate at 1 and 5 years of combined procedures in the subgroup.

### Definition

#### Technical success

Technical success of recanalization was defined as the complete elimination of HV or IVC obstruction and confirmed by venography. Technical success of TIPS was defined as successful placement of artificial stent between the hepatic vein and the portal vein. The stent position was confirmed by angiography, and the contrast medium flowed back into the right atrium smoothly through the intrahepatic shunt.

#### Clinical success

Clinical success of recanalization, combined procedures, and TIPS/DIPS was defined as an improvement of BCS related-symptoms and liver function after a technical success within day one to 90 days.

### Statistical analysis

The individual studies’ sample sizes were used to calculate a confidence interval (CI: 95%). The pooled measure and an equal weighted average rate were calculated in all participant studies. The data were pooled using a fixed effect model and random effect model. Heterogeneity between studies was assessed with the I^2^ and T^2^ tests (I^2^ > 50% or *P* ≤ 0.10 was considered statistically significant heterogeneity). Publication bias was estimated using Egger’s regression asymmetry test (*P* ≤ 0.05 represented statistically significant publication bias). Subgroup analyses were performed according to the continent of objectives. Statistical analyses were carried out using the R-version 3.5.3 software.

## Results

### Study characteristics

Overall, a total of 536 papers were identified in four databases. Among them, 71 original articles^9,14–83^ were eligible for systematic review and meta-analysis (Fig. 1). The general characteristics of the included studies are listed in Table 1. All included studies were published between 1995 and 2019. Among them, 33 (46.4%) were published between 2015 and 2019, and four (5.6%) before 2000. Most of the papers were published after 2010. Thirty-five (50%) studies were conducted in China, ten (14.2%) studies in India, four studies in the UK, three studies in Germany and Egypt, and two studies each in the USA, Italy, Netherland, and Turkey (Table 1).

**Table 1:** Overview on baseline of the included studies.

1st author/years of published/reference	Country	N.P.	M/F	Mean Age	Ste of stenosis			Type of treatment					Success rate		Re-Intervention		Survival rate	
					HV	IVC	Both	Recanalize	TIPS/DIPS	Stent	Angio	Thrombo	Technical (%)	Clinical (%)	Re-stenosis (%)	Dysfunction (%)	1 year (%)	5 years (%)
Fu YF 2015^22^	China	20	11/9	22–56	20	–	–	20	–	2	18	–	100	100	15	–	100	NA
Ding PX 2018^14^	China	108	69/39	25–74	–	1	107	107	–	13	94	12	99.1	99.5	16.5	–	95	86
Nagral A 2010^15^	India	11	5/6	4 m-11 y	11	–	–	5	6	2	3	–	100	100	0	–	90.9	NA
Rossle M 2004 ^16^	Germany	35	8/27	12–74	NA	NA	NA	–	33	–	–	–	94.2	100	57.5	57.5	91.4	91.4
Blum U 1995^17^	Germany	12	6/6	31–71	NA	NA	NA	–	12	–	–	–	100	83.3	41.6	41.6	75	NA
Pavri TM 2014^18^	USA	21/47	16/31	31–69	NA	NA	NA	–	21	–	–	–	100	85.7	57	52.3	100	81.5
Xu ke 1996^19^	China	32	6/26	20–56	12	20	–	31	–	17	20	–	100	96.8	37.5	–	96.8	96.8
Kathuri R 2014^20^	India	25	16/9	2–16	20	01	04	25	–	20	5	–	100	100	25	–	96	96
KhurooMS 2005^21^	Soudi arabia	16/40	17/23	15–64	16	19	–	6	8	–	6	–	87.5	92.8	14.2	62.5	92.8	92.8
Jagtap N 2017^23^	India	88	52/36	20–56	33	42	13	75	0/13	64	73	–	98.8	86.3	17.2	–	95.4	93.1
Zahan A 2010^24^	Germany	13	3/10	14–60	11	–	2	–	13	–	–	–	100	100	84.6	84.6	92.3	92.3
Zhou p-L 2017^25^	China	47	33/14	21–71	33	–	14	61	–	–	61	–	100	100	10.8	–	100	100
Yang F 2019^26^	china	33	16/17	44–74	–	33	–	33	–	15	18	–	100	100	9	–	100	100
Amara DN 2008^27^	India	38/49	24/25	1–57	29	10	10	22	15	22	2	–	97.5	100	16.2	–	94.5	94.5
Cheng D 2013^28^	china	141/145	90/55	10–82	45	8	92	133	1	16	133	48	95	100	4.4	–	99	NA
Fu Y-F 2015 ^29^	China	17	13/4	43–72	17	–	–	17	–	4	13	–	100	100	11.7	–	100	NA
Huang Q 2016^30^	China	265	131/134	18–79	–	–	265	263	–	56	263	–	99.5	100	14.6	–	99.6	98
Mishra TK 2003^31^	India	17	NA	30–50	NA	NA	NA	15	–	–	15	–	88.5	100	20	–	NA	NA
Mo A 2017^32^	Australia	27	11/14	21–76	NA	NA	NA	11	18	11	11	–	92.6	96	56	77.7	96	81
Zhang B 2013 ^33^	China	18	15/3	19–50	14	4	–	15	3	–	15	–	100	100	16.6	–	100	100
Meng X 2016^34^	China	55	39/14	NA	–	55	–	53	5	47	53	13	96.5	84.9	15	–	90	86
Chen ZK 2017 ^35^	China	68	39/29	22–52	68	–	–	68	–	8	60	–	100	95.6	27.9	–	96.9	93.4
Rathod K 2016^36^	India	190	102/88	15–55	147	40	3	84	106	84	78	–	100	80.5	10	10	100	100
Sang H-F 2014^35^	China	48	31/17	25–65	NA	NA	NA	43	–	31	43	5	89.6	100	9.3	–	100	100
Rosenqvit K 2016^38^	Sweden	13	6/7	16–63	NA	NA	NA	–	13	–	–	–	100	100	15.3	30.7	100	93
Bi Y 2018^39^	China	60	48/12	12–76	35	–	25	31	27	–	31	–	96.6	78	23.3	62.9	98.3	98.3
Darwish M 2009^9^	Netherland	64/163	70/93	16–83	NA	Na	N A	22	56	–	8	10	100	100	14	16	83	NA
Al-Warraky 2015^40^	Egypt	103	30/73	14–44	88	9	6	26	55/22	–	26	–	98	99	30.6	22.6	98	92
Eapen CE 2005^41^	UK	61	22/39	16–67	58	3	–	32	29	8	24	6	100	100	65.5	65.5	94	87
Li T 2009^42^	China	101	52/49	15–57	101	–	–	92	–	2	92	–	91	100	13	–	100	NA
Tripathi D 2014 ^43^	UK	67	21/46	15–70	NA	NA	NA	–	67	–	–	–	100	97	44.7	44.7	92	80
Fan X 2016^44^	China	60	27/33	18–60	51	–	9	27	33	–	27	–	100	96.6	13.3	13.3	96.6	96.6
Seijo S 2013^45^	Europe	70	NA	16–83	NA	NA	NA	8	62	–	8	9	100	94.2	0	–	84.2	84.2
Srinivas 2012^46^	India	12	7/5	28–55	–	12	–	12	–	5	7	–	100	100	8.3	–	100	100
Qiao T 2005^47^	China	44	25/19	19–77	8	32	4	45	–	45	–	–	93.1	100	8.5	–	100	100
Cheng D 2019^48^	China	162	94/68	18–78	–	–	162	157	–	35	208	47	96.9	92.9	8.2	–	100	NA
Tripathi D 2016^49^	UK	63	27/36	15–55	55	3	5	63	–	31	32	8	100	73	17.4	–	97	89
Sonavane 2018^50^	India	42	26/16	19–68	42	–	–	–	42	–	–	–	100	100	7.1	7.1	86	81
Zhang CQ 2003^51^	China	115	65/50	17–67	13	85	17	122	–	122	–	–	92.4	99.1	4.7	–	100	100
Hayek G 2016^52^	France	54	20/34	15–67	54	–	–	–	53	–	–	–	98	67.9	11.3	41.5	96	83
Bi Y 2018^53^	China	40	32/8	28–76	–	3	37	40	3	2	40	24	100	92.3	5.1	–	97.5	89.5
Bi Y 2018^54^	China	72	43/29	22–76	–	3	69	91	–	–	91	12	97.5	79.2	0	–	100	91.5
Ding PX 2019^55^	China	456	264/192	22–74	–	456	–	455	5	25	455	85	99.8	99.3	19.4	–	98.5	91.2
Shalimar 2017^56^	India	80	40/40	12–50	61	–	19	–	80	–	–	–	100	88.8	13.7	13.7	93.7	90
Ding PX 2015^57^	China	93	59/34	15–72	65	–	28	93		2	93	–	100	100	11.8	–	98.9	97.8
Darwish M 2007^58^	Netherland	17	10/6	19–50	16	–	11	–	16	–	–	–	94.1	94	0	62.5	80	72
Fu Y 2011^59^	China	18/29	13/16	23–67	4	18	–	22	–	–	22	–	100	100	5.5	–	100	100
Eldorry A 2011^60^	Egypt	25	9/16	14–57	NA	NA	NA	12	13	10	12	–	100	96	12	38.34	100	NA
Cheng DL 2018^61^	China	69	43/26	15–72	66	–	–	66	–	11	66	19	95.7	92.4	0	–	98.5	94
Yu C 2019^62^	China	56	30/26	29–65	–	56	–	55	–	–	55	–	98.2	100	12.7	–	100	100
Wu T 2002 ^63^	China	42	28/14	12–62	–	42	4	41	–	––	41	–	97.6	100	12.1	–	100	100
Han G 2013^64^	China	177	93/75	12–62	50	33	94	168	–	117	168	–	95	90	14.8	–	96	83
Fu YF 2015^65^	China	62	33/27	24–72	–	–	60	60	–	11	58	10	96.8	100	18.3	–	98.3	95
Cui Y-F 2015^66^	China	143	58/78	14–74	143	–	–	140	3	16	124	–	97.9	97.1	20.5	–	97.7	93.5
Boyvat F 2008^67^	Turkey	11	5/6	6–43	NA	NA	NA	–	11	–	–	–	100	81.8	45.4	81.8	100	NA
Kucukay F 2016^68^	Turkey	32	18/14	20–42	NA	32	NA	30	–	–	30	–	94	100	10	–	100	100
Lee BB 2006^69^	South Korea	17/28	13/15	28–68	2	26	–	15	2	6	15	–	100	82.3	23.5	–	100	NA
Griffith JF 1996^70^	UK	18	8/10	16–65	12	–	6	18	–	6	18	5	100	56	27.7	–	89	78
Cui YF 2015^71^	China	17	8/6	25–66	14	–	–	14		2	12	–	82.3	100	21.4	–	100	NA
Yang XL 1996^72^	China	42	28/14	16–56	–	38	–	38	–	–	38	–	91	100	2.6	–	100	100
Xue H 2009^73^	China	53	39/14	11–70	11	38	4	47	2	34	13	–	92.5	100	0	–	93.8	93.8
Molmenti 2005 ^74^	USA	11	5/6	22–78	NA	NA	NA	–	10	–	–	–	91	100	0	–	100	100
Garcia-pag 2008^114^	Italy	133	78/46	35–40	NA	NA	NA	–	124	–	–	–	93.2	82.2	49.1	49.1	95	87
Katerina 2013^115^	Greece	14	3/11	3–66	NA	NA	NA	–	14	–	–	–	100	100	28.5	28.5	100	100
Neumann 2013^76^	Denmark	14	3/11	17–66	NA	NA	NA	–	14	–	–	–	100	100	78.5	100	100	92.8
Wang R 2013^77^	China	29	NA	NA	–	29	–	28	–	18	–	15	96.6	100	14.2	–	100	100
Corso R 2008^78^	Italy	15	7/8	7–52	NA	NA	NA	–	15	–	–	15	100	100	40	40	86.6	86.6
Ding PX 2010^79^	China	13	9/4	39–74	–	13	–	13	–	––	13	13	100	100	0	–	100	NA
Fu YF 2015^80^	China	66	34/32	21–79	66	–	–	66	–	18	50	–	100	100	16.6	–	100	100
Mukund A 2018^82^	India	136	96/40	1–67	106	30	–	92	44	64	92	4	100	87.5	5.1	5.1	94.1	94.1
Mohamed 2018^83^	Egypt	118	43/75	20–45	118	–	–	–	118	–	–	–	100	83.0	40.74	40.74	95.8	91.5

A total of 4407 patients underwent endovascular intervention procedures. Among them, 98.9% of patients were considered technical successes and 96.9% achieved clinical improvement. The site of obstruction was documented in 53 (75.7%) studies, including 42.25% in HV, 30.98% in the IVC, and 26.76% in combined (HV and IVC) (Table 1). In subgroup analysis, recanalization was used in 31(43.66%) studies, combined procedures (recanalization and TIPS) in 23 (32.39%) studies, and TIPS in 17 (23.94%) studies (Table 1).

### Study quality assessment

Patients were consecutively admitted in 57 (80.28%) studies^9,12,14–25,28,30–39,41–45,47–57,59,64,66,68,70–75,78–83^. Fifty one (71.83%) studies were considered to be of high-quality^9,12,14,16–19,21–23,25,26,31–34,39,41–43,45–47,49–56,58,59,61–64,66–68,70–75,78–83^ and six (8.45%) studies were of poor-quality^13,15,29,65,76,77^. The site of obstruction was clearly reported in 53 (75.7%) studies (Table 1). The interval of enrollment and eligibility criteria were recorded in all included studies. All patients were diagnosed with BCS and treated with endovascular intervention procedures.

### The technical success rate of endovascular intervention procedures

**Figure 2:**
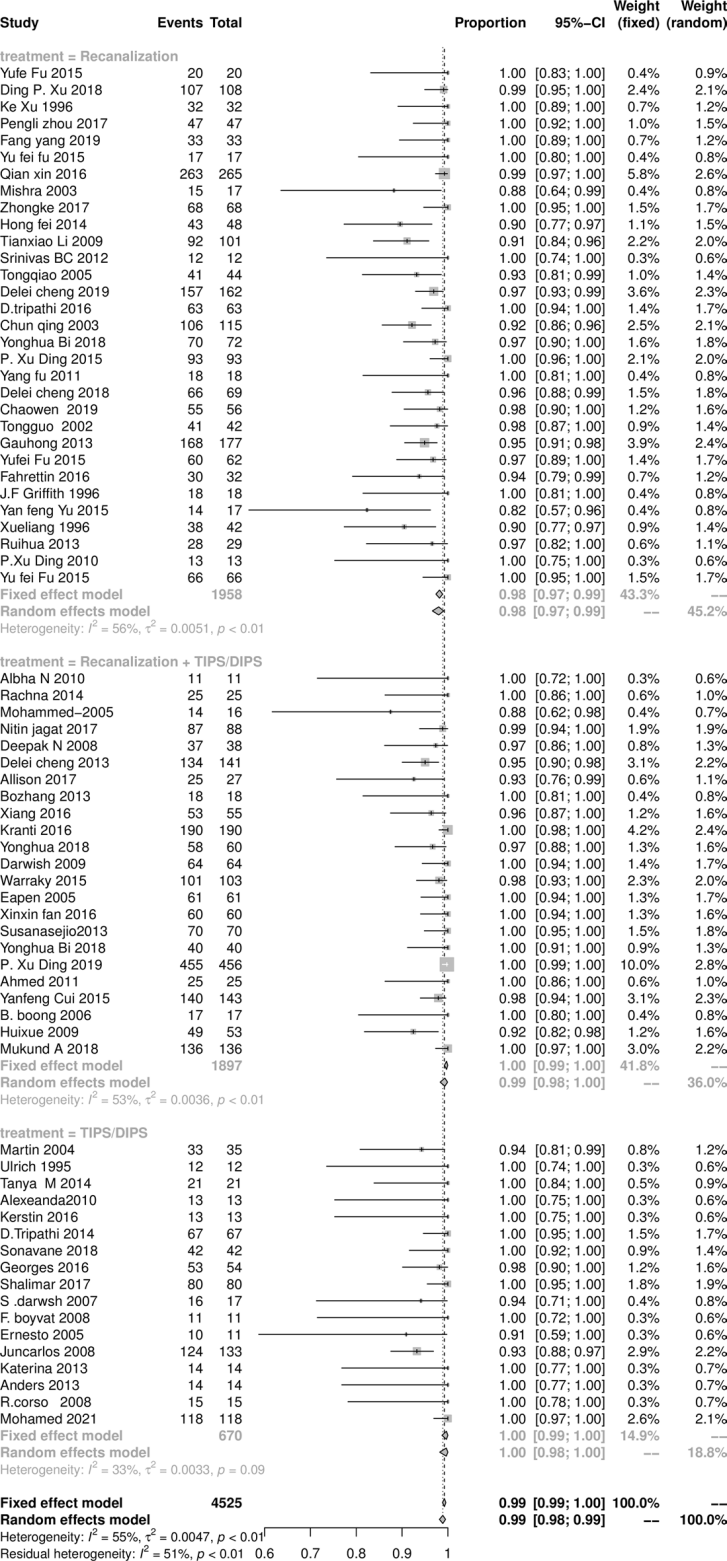
The Forest plot of technically success rate of intervention procedures in BCS patients, horizontal lines indicate 95% confidence intervals, square size indicates study specific statistical weight, and diamond indicates the overall treatment effect with 95% confidence intervals.

The technical success rate of all individual studies is shown in Fig. 2.The pooled result of total technical success procedures was 98.9% (95% CI 97.8‒98.9%), with statistically significant heterogeneity among studies (*I*^2^ = 54%, *P* < 0.01). The pooled results of the recanalization, combined procedures, and TIPS subgroups were 97.9% (95% CI 96.8‒98.9%), 98.9% (95% CI 97.9‒99.9%), and 99.8% (95% CI 97.9‒99.9%), respectively.

### The clinical success rate of endovascular intervention treatment

**Figure 3:**
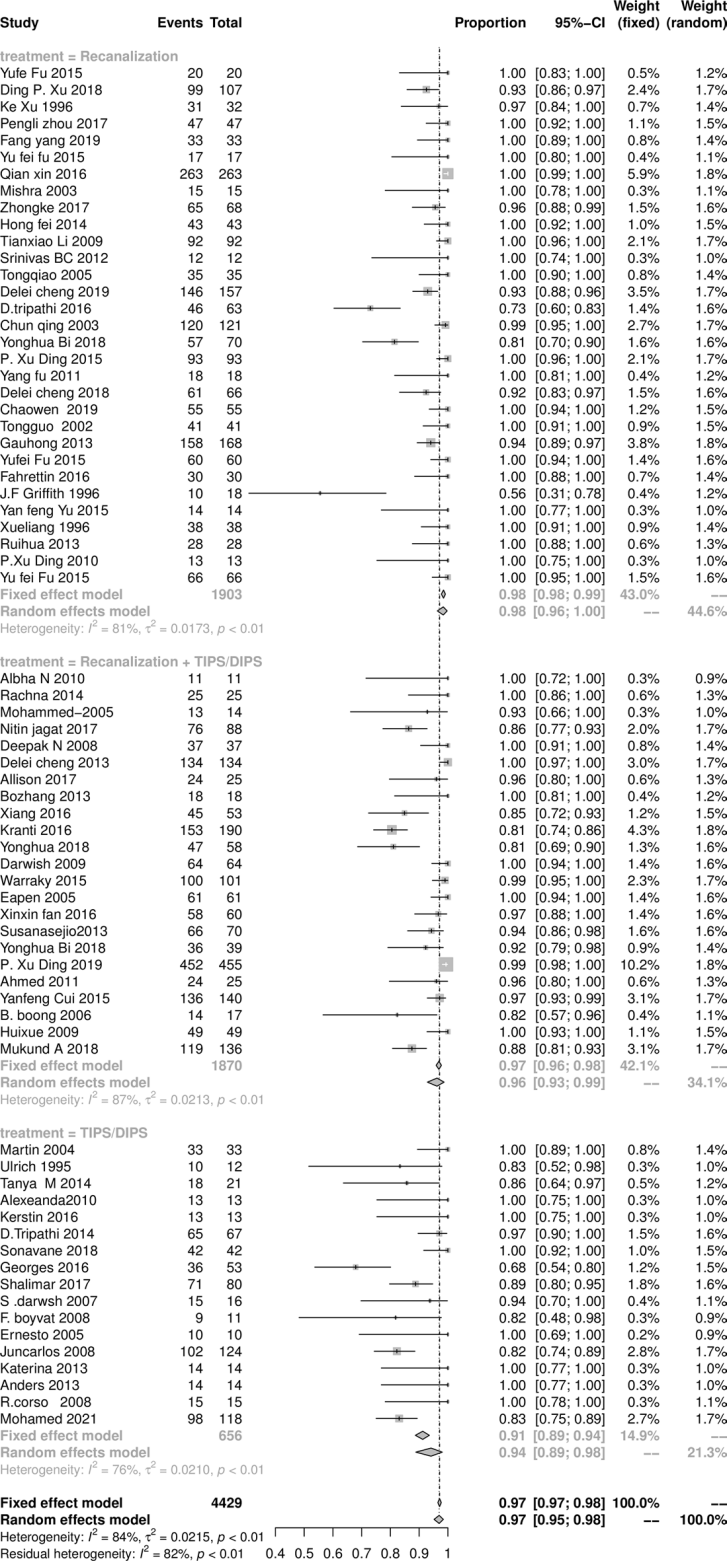
The Forest plot of clinically success rate after intervention treatment in BCS patients, horizontal lines indicate 95% confidence intervals, square size indicates study specific statistical weigh, and diamond indicates the overall treatment effect with 95% confidence intervals.

The clinical success rates of all cases of BCS are shown in Fig. 3. The pooled result of the total patients with a clinical success rate was 96.9% (95% CI 94.9‒98.9%), with statistically significant heterogeneity among studies (*I*^2^ = 83%, *P* < 0.01). The pooled results of the recanalization, combined procedures, and TIPS subgroups were 97.9% (95% CI 95.9‒99.9%), 95.6% (95% CI 92.7‒98.9%), and 94.0% (95% CI 88.5‒98.8%), respectively.

### The rate of re-intervention at 5 years after initial intervention treatment

**Figure 4:**
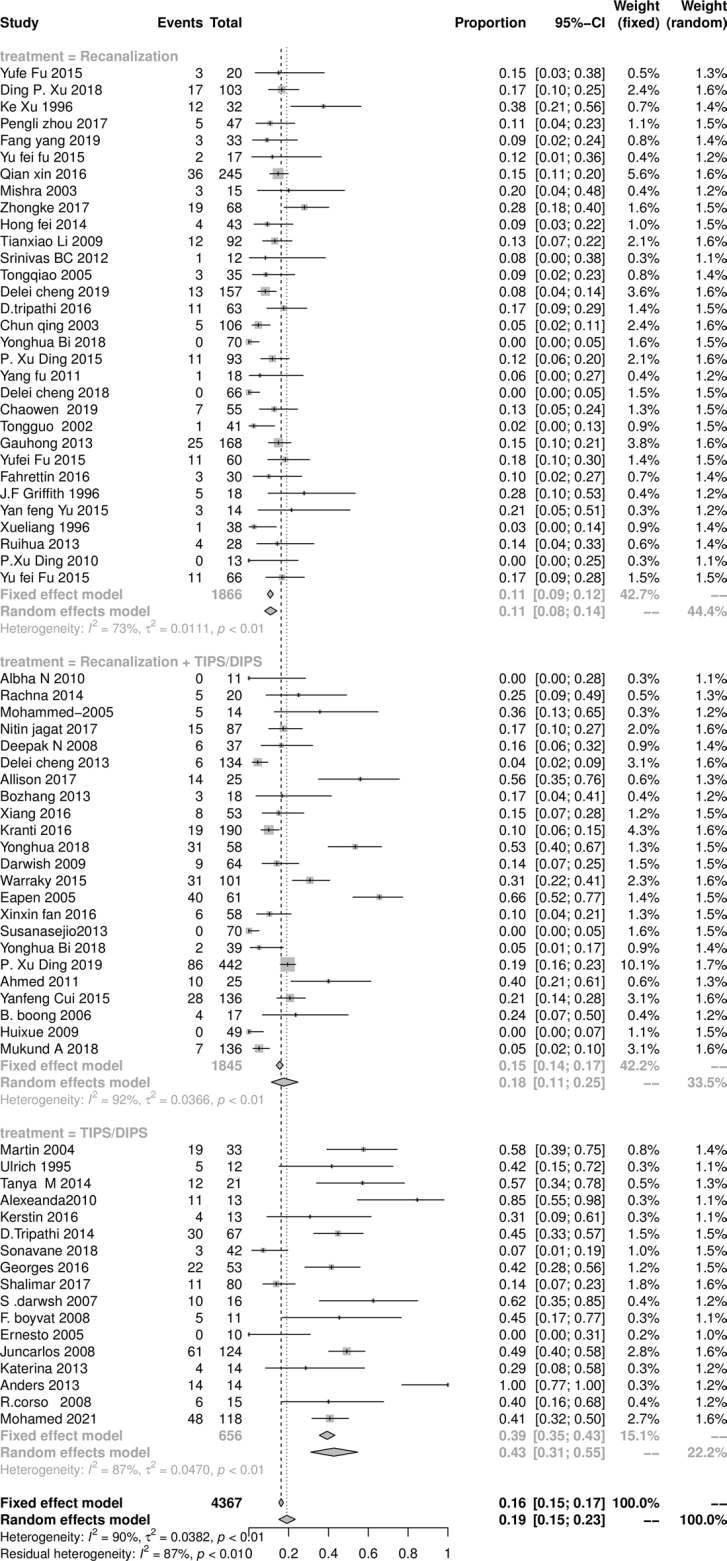
The Forest plot of the re-intervention rate after initial intervention procedures in BCS patients, horizontal lines indicate 95% confidence intervals, square size indicates study specific statistical weigh, and diamond indicates the overall treatment effect with 95% confidence intervals.

The vascular re-occlusion, stent stenosis, and shunt dysfunction at 5 years after initial endovascular intervention procedures of BCS are shown in Fig. 4. The pooled result of total re-intervention was 18.9% (95% CI: 14.7‒16.9%), with statistically significant heterogeneity among studies (*I*^2^ = 90%, *P* < 0.01). The pooled results of the recanalization, combined procedures, and TIPS subgroups were 10.8% (95% 7.5‒13.8%), 17.9% (95% CI 10.9‒24.9%), and 42.9% (95% CI 29.9‒56.8%), respectively.

### The survival rate at 1 and 5 years after endovascular intervention procedures

**Figure 5:**
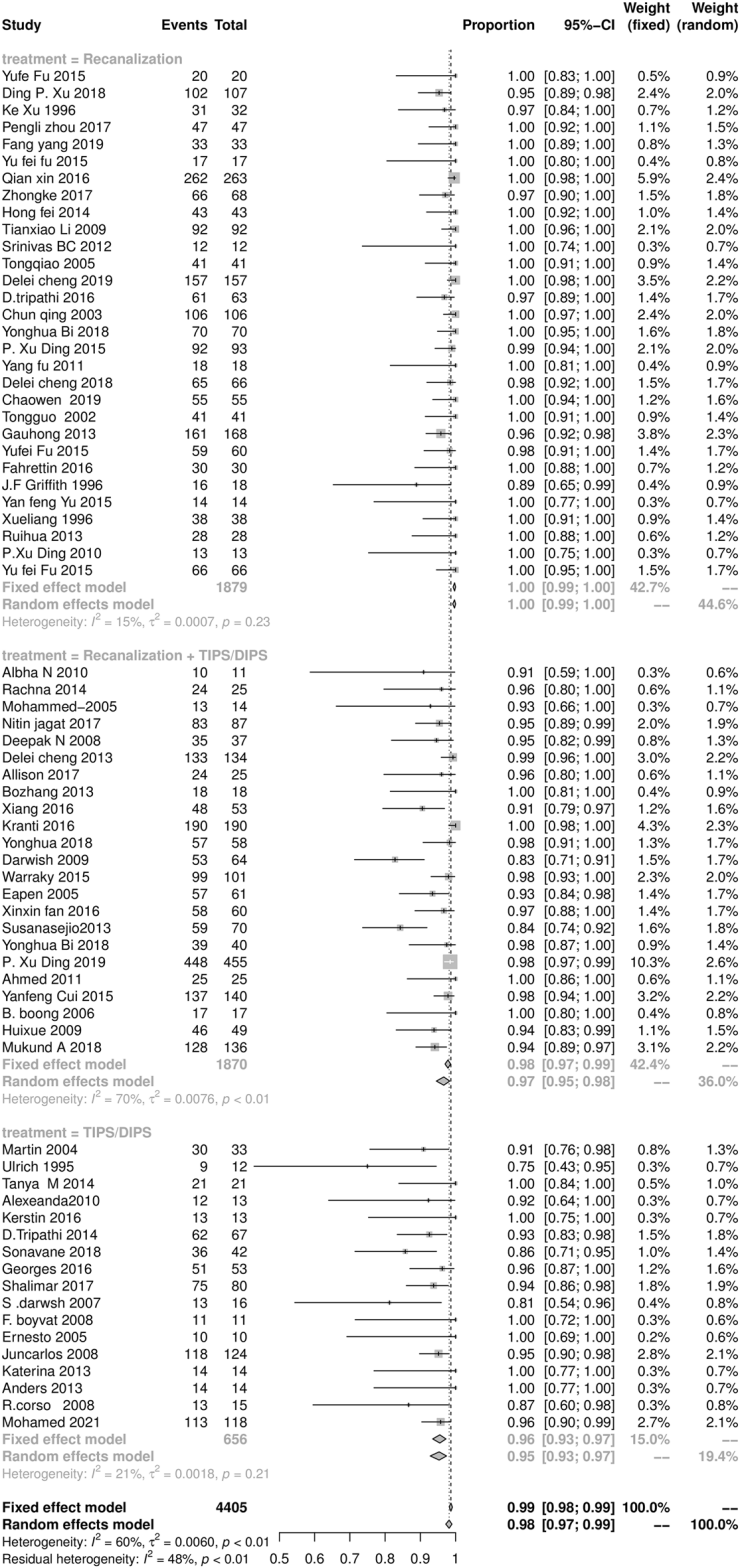
The Forest plot of the survival rate at 1 year after initial intervention procedures in BCS patients, horizontal lines indicate 95% confidence intervals, square size indicates study specific statistical weigh, and diamond indicates the overall treatment effect with 95% confidence intervals.

**Figure 6:**
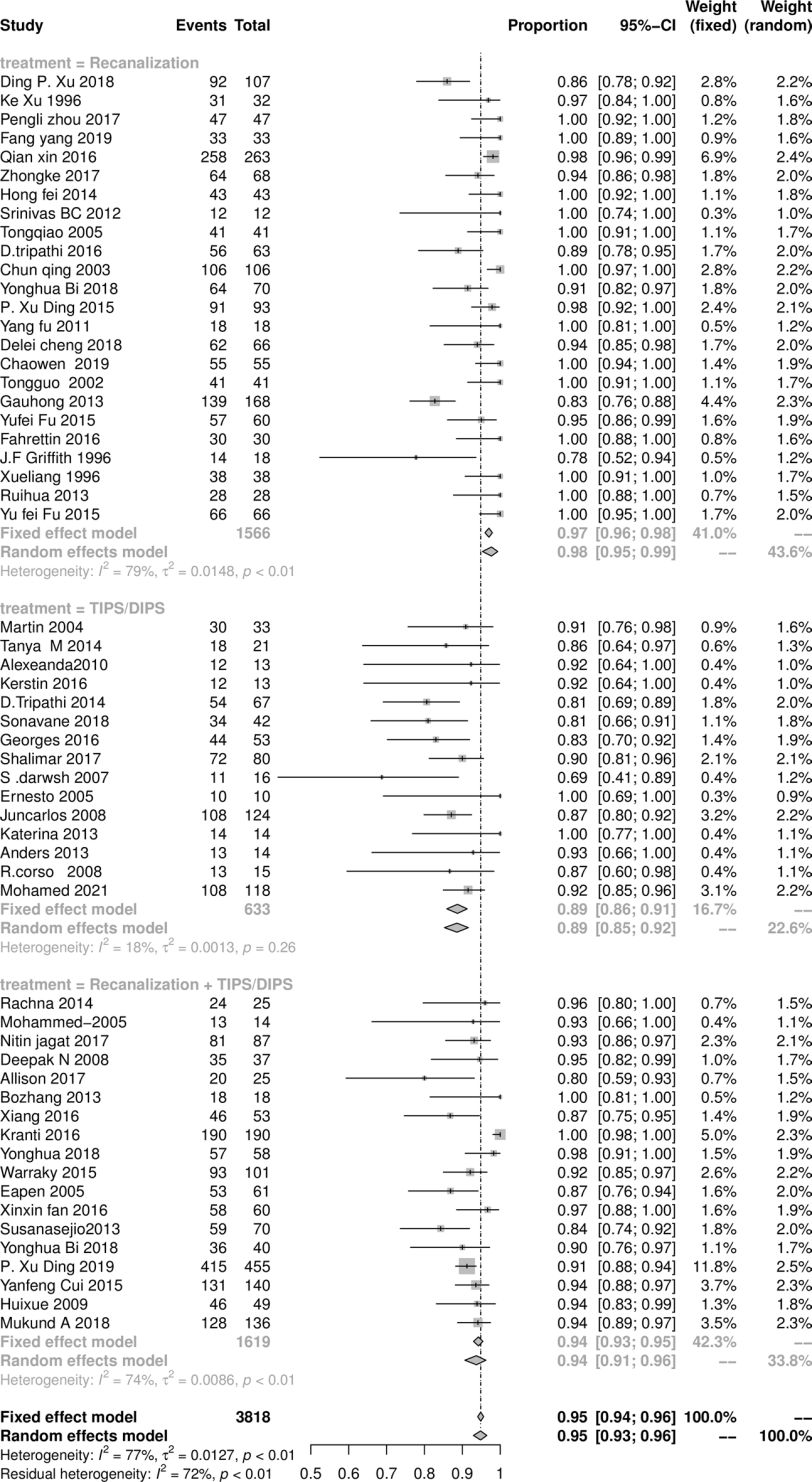
The Forest plot of the survival rate at 5 years after initial intervention procedures in BCS patients, horizontal lines indicate 95% confidence intervals, square size indicates study specific statistical weigh, and diamond indicates the overall treatment effect with 95% confidence intervals.

The survival rate of endovascular intervention therapy of BCS patients at 1 and 5 years after initial intervention procedures are shown in Figs. 5 and 6. The pooled result of the total survival rate at 1 year was 98.9% (95% CI 96.8‒98.9%), with statistically significant heterogeneity among studies (*I*^2^ = 60%, *P* < 0.01). The pooled results of the recanalization, combined procedures, and TIPS subgroups were 99.9% (95% CI 98.9‒99.9%), 96.9% (95% CI 94.8‒97.9%), and 94.9% (95% CI 91.9‒96.7%), respectively. Similarly, the pooled result of the total survival rate at 5 years was 94.9% (95% CI: 92.5‒96.9%), with statistically significant heterogeneity among studies (*I*^2^ = 77%, *P* < 0.01). The pooled results of the recanalization, TIPS, and combined procedures subgroups were 97.9% (95% CI 94.8‒98.9%), 88.9% (95% CI 84.9‒91.9%), and 93.9% (95% CI 90.9‒95.9%), respectively.

### Publication bias

The results of publication bias in the studies evaluated with Egger’s test. The publication bias for the technical success rate of endovascular intervention procedures (*P* = 0.0335), clinical success (*P* = 0.5567), re-intervention (*P* = 0.08108), the survival rate in one year (*P* = 0.01549) and the survival rate at five years (*P* = 0.8909). Although the *P* value of technical success and survival rate at 1 year was statistically significant.

## Discussion

This extensive study evaluates and updates the clinical efficacy and long-term outcome of endovascular therapy in BCS patients and compares recanalization, TIPS/DIPS, and combined procedures. The technical and clinical success rates were 98.9% and 96.9%. After the initial endovascular treatment, the re-intervention rate was 18.9%, and the survival rates at 1 and 5 years after the initial endovascular treatment were 97.9% and 94.9%, respectively. The findings indicate that endovascular intervention treatment is safe, effective, and provides long term survival rates in patients with BCS.

Most of the studies were conducted in Asian countries, half of the study sample was from China (50%), and 45.7% of the study sample was published from 2015 to 2019. Most of the patients were treated with endovascular recanalization with or without stent placement. The subgroups’ pooled result showed that the re-intervention treatment rate was high in TIPS/DIPS, the technical success rate higher in combined procedures, and the clinical success rate and the survival rate at 1 and 5 years were higher with recanalization. It was interesting to find that the most common obstruction site was HV in the Asian countries. Also, most Asian studies reported the most common obstruction sites IVC and combined (HV and IVC)^48,84,85^. However, some studies have reported HV obstruction as the most common cause of BCS in the Asian population^27,86^.

BCS can be classified according to etiology (primary and secondary), site of obstruction (HV, IVC, and combined HV + IVC), the manifestation of the disease (fulminant or non-fulminant), and duration of the disease (acute, subacute or chronic)^2^. The clinical presentation is highly variable but may be categorized as acute/fulminant hepatic failure, as subacute without evidence of cirrhosis and as chronic with evidence of portal hypertension and cirrhosis^87^. In this meta-analysis, we found most of the studies were treated according to the site of obstruction (42.25% in HV, 30.98% in the IVC, and 26.76% in combined HV + IVC). Recanalization and TIPS treatments for BCS depend on the anatomical site and the extent of obstruction and liver function^58^. HV recanalization and TIPS have become the main treatment for HV-type BCS ^16,33,38^.

BCS is a rare disorder and therefore management guidelines are based on the retrospective case series, expert opinion and clinical presentation^75,88–90^, due to the lack of randomized controlled trials study^9^. BCS is more prevalent in developing countries such as, China, India, Nepal and South Africa. In contrast, the most common cause is membranous obstruction, an underlying thrombotic disorder that has been in only a few patients^28^, where the treatment choice is recanalization. However, only 29–41% of Western patients have membranous or segment obstruction^41,91^, and pure hepatic vein thrombosis accounts for more than half of BCS cases^92^. In contrast, recanalization is not applicable in most Western patients with BCS, and TIPS is a preferable treatment^41^.

Membranous obstruction of IVC is a common cause of hepatic venous outflow obstruction, which has short web narrowing to a long segmental occlusion with or without narrowing of hepatic vein^46,93^. In the West, HV thrombosis is the most common cause, while in Asian countries isolated IVC membranous webs are more common^84,85^, and two-thirds of IVC obstructions are due to membranous or segment obstruction. The long-term treatment outcome of endovascular intervention treatment was better for membranous obstruction of IVC rather than segmental obstruction of IVC. PTA alone could be the optimal treatment for membranous obstruction and stenting should be more strongly recommended for a segment of obstruction of IVC^30^.

The thrombophilic factors are responsible to development of BCS, which is detected in up to 84% of BCS patients^10,11^. The most common thrombophilic factors are myeloproliferative disease and factor V linden^11^. In over 25% of BCS cause more than one thrombophilic state may be present with BCS patients^94^. Most inherited thrombophilias result increased thrombosis due to an impaired neutralization of thrombin or failure to control of generation of thrombin^95^. Data show that prothrombotic disorders are not common in china as a cause of unknown factors in Chinese BCS patients^96^. The thrombophilia is more commonly found in western BCS patient than Chinese BCS patients^97^.

HV recanalization was performed in patients with short-segment HV obstruction (< 3 cm), and stenting was performed in long segment HV occlusion (> 3 cm) with large collateral vein drainage^36^. HV recanalization is usually difficult for BCS patients with segmental obstruction, whereas TIPS placement has been widely used for BCS patients who fail to HV recanalization^41,98^. In patients with compensatory but obstruction accessory hepatic vein (AHV), Fu et al.^22^ reported that recanalization of the AHV is a simple, safe, and effective treatment option for long segmental obstruction of the HV. However, TIPS is often the treatment choice for long segmental obstruction of HV^41,76^.

In Western countries, where HV extensive thrombosis is more common mostly due to myeloproliferative neoplasm^92,99^, TIPS placement is used to treat most patients. In Asia, where HV obstruction is mostly due to membranous webs^84^, recanalization (PTA and stenting) is a more common treatment. In this extensive meta-analysis, TIPS placement was more used in Western countries than Asian countries, and membranous webs had better outcomes than extensive thrombosis.

The step-wise therapeutic algorithm of BCS includes medical therapy with anticoagulant drugs and thrombolysis—recanalization with or without stent placement—TIPS/DIPS and liver transplantation^45,100^. However, due to poor long-term medical therapy outcomes, most of the studies used recanalization with or without stent placement as the first-line treatment for BCS^14,15,22,26,35,59,80^. Moreover, TIPS was used in circumstances of failed recanalization, refractory ascites, portal hypertension, variceal bleeding, and long segment obstruction or diffused obstruction of the HV^21,24,41,43,52^.

Recanalization is a physiological procedure that maintains the natural blood flow in HV/IVC^33,36,41^. It can minimize the risk of hepatic encephalopathy, and remains a first-line treatment option for BCS patients^35,61^. However, TIPS has less portal vein blood perfusion in the liver than recanalization and a high risk of hepatic encephalopathy due to the formation of a blood ammonia level and impaired liver function after shunt placement^19^. The secondary patency of recanalization with angioplasty + stent (79% and 92%) was higher than recanalization with only angioplasty (64% and 69%) at 1 and 5 years^49^. The treatment of BCS with an expandable metallic stent was introduced to decreasing the re-stenosis rate after angioplasty^101^. This study found that most studies adopted recanalization (44.28%) as a first-line treatment because it is a relatively simple and quick procedure. Also, the risk of hepatic encephalopathy after recanalization is lower than TIPS/DIPS. TIPS/DIPS has only been applied as an alternative treatment option for selective cases of BCS, but it may have a high risk of complication after shunt implantation^49,102^. However, several previous studies have reported the high patency rate and long-term outcome of TIPS/DIPS for BCS^43,75,103–106^. Liver transplantation is a second surgical option for BCS when a rapidly progressive liver failure occurs before or after TIPS^107,108^.

In this meta-analysis, we found that the survival of recanalization and TIPS were 99.9% and 94.9% at 1 year and 97.9% and 87.9% at 5 years, respectively. The survival of patients in this study seems comparable to that of a previous meta-analysis Zhang et al.^109^, which showed the survival of recanalization and TIPS were 95.9% and 87.3% at 1 year and 88.6% and 72.1% at 5 years, respectively. Tripathi et al.’s^49^ retrospective study showed the survival of recanalization and TIPS were 97% and 88% at 1 year, 89% and 79% at 5 years, and 85% and 73% at 10 years, respectively. Garcia-pagan et al.^75^ reported that the survival of TIPS with liver transplantation at 1and 5 years were 88% and 78%, respectively. Mentha et al.^110^ reported that survival of liver transplantation for BCS at 1, 5, and 10 years were 76%, 71%, and 68%, respectively. Nonetheless, our meta-analysis results indicate a progressive improvement in survival rate with endovascular therapy for BCS treatment.

Our results show that recanalization therapy had a better prognosis than TIPS therapy. Similarly, the prognosis of recanalization was shown by previous meta-analyses^109^. Mukund et al.^82^ reported that BCS patients treated with recanalization have improved biochemical profile and overall outcome relative to DIPS treatment. However, the survival and clinical improvement were similar in both groups, and Tripathi et al.^49^ also reported no significant difference in the results of patients treated with recanalization and TIPS.

Recently, endovascular intervention treatment has emerged as an advanced therapeutic option for BCS patients. The TIPS/DIPS procedures have rapidly replaced the traditional surgical shunt due to minimal invasiveness, less blood loss, low infection rate, quick recovery, shorter hospital stay, and increased long-term survival rate^9,24^. The technical success rate of TIPS in BCS has been reported to be between 75 and 100%. Shunt dysfunction at 5 years ranges between 40 and 75%, and the survival rate at 1 and 5 years after the initial intervention treatment was 85% and 75%, respectively^16,24,74,111,112^. It was found that the TIPS/DIPS technical success rate was 98.9%, while shunt dysfunction was 42.9%, and the survival rates at 1 and 5 years were 94.9% and 87.9%, respectively.

The development of new techniques and improvements in radiological intervention has established endovascular intervention therapy as a treatment of choice for BCS patients. This method provides an effective treatment modality for BCS patients and prevents progression to life threatening conditions, such as portal hypertension and other related complications^47,113^.

In this updated analysis, most of the included study was original articles published after 2010. The survival rates at 1 and 5 years were 97.9% and 94.9%, the success rate of operation was 98.9%, and the re-intervention episode was 18.9%. Similarly, the survival rates of recanalization, combined procedures, and TIPS/DIPS in BCS at 1 and 5 years were 99.9%, 96.9%, and 94.9% and 97.9%, 93.9%, and 87.9%, respectively. Publication bias of technical success (*P* = 0.0335), clinical success (*P* = 0.5567), re-intervention (*P* = 0.08108), the survival rate at 1 year (*P* = 0.01549) and survival rates at 5 years (*P* = 0.8909) were observed. The patients with recanalization treatment had a better prognosis and outcome than the combined procedures and TIPS/DIPS treatment. Additionally, the clinical success rate, shunt dysfunction rate, combined procedures, and obstruction site were analyzed. Overall, comparatively the statistical results are progressively more favorable than the previous study^109^.

Despite the latest update on the role of endovascular intervention therapy for BCS, the present study has several limitations: First, studies on endovascular intervention therapy for BCS worldwide are limited. Retrievable articles were available between 1995 and 2019. Most of the relevant studies were published between 2015 and 2019 and only four studies were published before 2000. Second, some articles were excluded during the selection because of a lack of information about re-intervention and long-term survival rates. Third, there was an unequal distribution based on studies conducted in different geographical regions. Most of the study samples were from Asian and European countries; the African and American data were scarce. Also, some studies were excluded due to low study quality.

## Conclusion

The systematic review and meta-analysis findings further solidify the role of endovascular intervention treatment in BCS as safe and effective. It maintains high technical and clinical success, and long-term survival rates. The recanalization treatment had a better prognosis and outcome than the combined procedures and TIPS/DIPS treatment. The endovascular intervention procedures are the preferred first-line treatment in selected patients with BCS. However, randomized controlled multidisciplinary centers studies are needed to further evaluation.

## Supplementary Information

Supplementary Table 1.

## Data Availability

The data presented in this study are available on request from the corresponding author. The data are not publicly available due to legal restrictions.
